# Disentangling the Effect of Sex and Caregiving Role: The Investigation of Male Same-Sex Parents as an Opportunity to Learn More About the Neural Parental Caregiving Network

**DOI:** 10.3389/fpsyg.2022.842361

**Published:** 2022-02-14

**Authors:** Michele Giannotti, Micol Gemignani, Paola Rigo, Alessandra Simonelli, Paola Venuti, Simona De Falco

**Affiliations:** ^1^Department of Psychology and Cognitive Sciences, University of Trento, Rovereto, Italy; ^2^Department of Developmental Psychology and Socialization, University of Padua, Padua, Italy

**Keywords:** parental brain, parental involvement, same-sex parents, parenting, neural, caregiving

## Introduction

Since the traditional family structure (i.e., a mother, a father, and children) does not reflect the composition of families in contemporary society anymore, research has gradually expanded to examine parenting and child development in new family forms (Carone et al., 2021). Additionally, numerous studies have pushed beyond the study of mothering, considering paternal role as a remarkable factor (Singley et al., 2018). In this regard, recent data has described increasing father involvement in childcare over decades, pointing to its positive effect on the health of children and parents (Lamb, 2010). In this framework, the inclusion of male same-sex couples in the field of parenting research has provided further insights in terms of understanding the influence of caregiving role on paternal outcomes and family dynamics (Brown et al., 2012; Allport et al., 2018). As a general remark, research displayed that male same-sex parents showed positive parenting qualities (i.e., high level of warmth and responsiveness, great number of interactions, and low level of disciplinary aggression) (Golombok et al., 2014; Baiocco et al., 2015; Feugé et al., 2020), and suggested that parenting quality and child outcomes are the result of the family processes (i.e., warmth, sensitivity) rather than of the family structure (Farr and Vázquez, 2020; Carone et al., 2021). However, only few results regarding the neurobiology of human caregiving in male same-sex parents have been outlined so far (Abraham et al., 2014). Noteworthy, neurobiological characteristics of parenthood would provide a powerful theoretical and empirical framework in understanding reciprocal interactions between caregivers and infants (Swain, 2011). In this article, we initially propose a summary of evidence addressing functional neurobiological aspects of fatherhood in different-sex families, since no inherent differences in the parental caregiving network (i.e., brain structures and activations supporting parental caregiving) has been described as a function of sexual orientation (Abraham et al., 2014). Next, we review some findings on the relation between neurobiological activations and paternal involvement, and we highlight the potential opportunities and challenges of conducting research on male same-sex parents specifically. Besides enriching the conceptual grounding, investigating the neurobiological characteristics of male same-sex parents in the light of their involvement in childcare could improve the soundness of studies in methodological terms, by disentangling biological (i.e., sex) and socio-cultural factors (i.e., paternal involvement) influencing paternal neural responses to infants.

## Paternal Brain Circuits in Different-Sex Families

Research on animal models has pointed out that paternal caregiving may rely on some specific neural circuits and networks when compared to those underlying maternal behaviors (Swain et al., 2014; Rilling and Mascaro, 2017). In spite of a potential overlap between maternal and paternal networks related to caregiving, human fathers have shown a specific pattern of brain activations when responding to infant stimuli (Atzil et al., 2012; Abraham et al., 2014). Generally, whilst mothers tend to activate an emotional neural circuitry of subcortical structures (i.e., Amygdala, Nucleus Accumbens, Insula and Ventral Anterior Cingulate Cortex), fathering behaviors mostly rely on the activity of socio-cognitive cortical areas (i.e., Medial Prefrontal Cortex, Dorsolateral Prefrontal Cortex, Dorsal-Anterior Cingulate Cortex, Superior Temporal Gyrus and Inferior Frontal Gyrus) (Atzil et al., 2012; Rajhans et al., 2019). Summarizing findings related to paternal brain responses to auditory and visual infant stimuli, Provenzi et al. (2021) described three main brain networks. Specifically, the activation of Mentalization-related areas (i.e., Superior Temporal Sulcus, Medial Prefrontal Cortex) was associated with fathers responding to infant stimuli, in order to appropriately understand feelings and thoughts. Similarly, the activation of an Embodied Simulation network (i.e., Anterior Insula, Middle and Lateral Superior Frontal Gyrus, Ventral Anterior Cingulate Cortex) have been found to promote an understanding of infants' intentions by simulating internal feeling states, and an Emotional Regulation network (i.e., Inferior Frontal Gyrus, Orbitofrontal Cortex) have been proposed to foster sensitive caregiving by strengthening emotional processes. It is notable that these hard-wired functional brain networks, in turn, are associated with hormonal changes modulating fathering behaviors (Storey et al., 2020).

## Paternal Brain and Involvement in Childcare in Different-Sex Families

Beyond the sex-specificity of some neurobiological patterns (De Pisapia et al., 2013; Rigo et al., 2017; Rajhans et al., 2019), it has been suggested that the activations of the parental caregiving network may not be only ascribable to biology-related factors (i.e., sex), but also influenced by a great variety of other variables (i.e., contextual requirements, role definitions, cultural beliefs, and individual life histories) (Feldman et al., 2019). Accordingly, the neural network related to parenting proved malleable to adapting to social environments and childcare experiences (Horstman et al., 2021). In line with a previous review (Storey et al., 2020), we suggest that even the endocrine patterns related to fatherhood could be regarded as plastic and flexible. Therefore, the consideration of relevant contextual factors such as fathers' involvement might be essential for a better understanding of the whole paternal neurobiology. Relatedly, neurobiological research reported a relationship between greater paternal involvement and the activations of some brain areas involved in the caregiving network (Feldman, 2015), namely a larger activation of the Ventral Tegmental Area and a moderate level Anterior Insula activity (Mascaro et al., 2013, 2014). Also, more hours spent in direct childcare has been related to higher Amygdala resting-state functional connectivity with other parenting related brain areas, such as Supramarginal Gyrus, Postcentral Gyrus, and Superior Parietal lobe (Horstman et al., 2021). Recently, fathers' self-reported attitude toward their role, which may be considered as one trait-like predictor of father involvement, was positively associated with the degree of interpersonal neural synchronization (INS) in father–child interaction (Nguyen et al., 2021). The amount of fathers' involvement has been additionally correlated with the regulation of hormones triggering caring behaviors, such as a downregulation of Prolactin and an upregulation of Cortisol (Gettler et al., 2011; Kuo et al., 2018). In line with evidence from animal models (Featherstone et al., 2000; Nunes et al., 2001; Swain et al., 2014; Storey and Ziegler, 2016), assuming the role of a committed parent and engaging in active care of the offspring may modulate parental responses toward infants, promoting a sensitive caregiving. However, a study has recently outlined that the investigation of parental involvement has been highly debated in both methodological and conceptual terms (Chen and Zhu, 2017), since some measures did not prove sufficiently appropriate for capturing all the nuances of that complex construct. For instance, some authors considered only partial components of paternal involvement, or collapsed heterogeneous aspects into a limited measure (Chen and Zhu, 2017). As an additional issue, the gendered division of childcare in heterosexual couples make it difficult to disentangle the difference between the role of sex and involvement. Considering statistical analyses, this could result in a systematic bias, since fathers may be consistently less involved as compared to mothers. To appropriately disambiguate this bias, we suggest that including male same-sex parents could unravel the actual contribution of caregiving involvement to paternal responsiveness at neurobiological level, thereby excluding the confounding role of socio-cultural differences related to the biological sex of parents. As compared to same-sex mothers, the amount of contact with children may be particularly relevant for male same-sex parents' neurobiology, since they do not experience the physiological changes that come along with gestation.

## Neurobiology of Parental Caregiving in Male Same-Sex Parents

To date, only one study (Abraham et al., 2014) addressed the neurobiological correlates of male same-sex parents when responding to videos of parent-infant interactions. Particularly, authors tested heterosexual different-sex couples comprising primary-caregiving mothers (PC-Mothers) and secondary-caregiving fathers (SC-Fathers), and primary-caregiving homosexual fathers (PC-Fathers). Results showed that PC-fathers displayed an Amygdala activation as high as PC-Mothers, and a Superior Temporal Sulcus activation as high as SC-Fathers. Additionally, PC-fathers showed a significant functional connectivity between the two brain structures (i.e., Amygdala, Superior Temporal Sulcus) in response to self-infant interactions. Even though the task-related functional connectivity between Amygdala and Superior Temporal Sulcus was observed only in PC-fathers, the overlap between the two structures was linked to the time spent in infant care among all fathers. Importantly, no difference emerged between biological and adoptive homosexual fathers in behavior, oxytocin concentrations or the extent of activations in any brain areas, thus highlighting the important role of involvement in childcare over other factors. Overall, being engaged with childcare may be associated with the activation of a global caregiving network involving both cortical and subcortical brain areas subserving parenting, in women and men and in both biological parents and those genetically unrelated to the child (Abraham et al., 2014). This complex coupling between emotion and cognition networks may ultimately promote a sensitive parenting. In light of these results, we might confirm that the investigation of paternal involvement and brain responses to infant cues among male same-sex parents would provide some valuable insights into the distinction of the role of sex and involvement. This could be particularly useful, especially when considering that the division of childcare in same-sex couples is more egalitarian as compared to that of heterosexual couples (Tornello et al., 2015; Rubio et al., 2020). In fact, whilst same-sex parents might perceive more equity in terms of childcare division, mothers usually spend more time with childrearing as compared to fathers in different-sex families (Geist and Cohen, 2011; Fossoul et al., 2013; Feugé et al., 2019). Additionally, gay fathers displayed high levels of involvement for both physical play and emotional support domain, being close to the traditional paternal role with regard to physical play but standing out with high levels of involvement in emotional support as well (Feugé et al., 2019).

Instead, it is reasonable that results investigating the impact of involvement in fathers from different-sex families may be biased by traditional gender norms and values, and so inclusive research of different family forms is needed, especially those in which fathers are the primary caregivers (Ellis-Davies et al., 2022). Despite the limited amount of neurobiological research, a slightly larger number of behavioral studies addressed paternal sensitivity focusing on male same-sex parents (e.g., Feugé et al., 2020). Some of these studies (Carone et al., 2020; Ellis-Davies et al., 2022) have not succeeded in finding significant associations between involvement and parenting qualities in fathers, as they may have been limited by the way caregiver role has been measured. For instance, as compared to an absolute and continuous measure of caregiving involvement, a relative measure of the construct (i.e., the degree to which fathers and mothers are involved in child rearing activities as compared to their partners) has failed to detect a significant effect also in another relevant study (Helmerhorst et al., 2022). Promising findings in this field have been outlined by Abraham et al. (2014), with results showing that primary-caregiving fathers displayed a greater dyadic synchrony than secondary caregivers, thereby highlighting the role of involvement for the quality of parent-child interactions. Notably, this study adopted an appropriate and detailed measure to capture the nuances of paternal involvement, and this sound methodology might be linked to the overall encouraging findings. Considering the link between the behavioral and neurobiological characteristics of fathers, it is notable that these results could be seen as promising for future research addressing underlying parental neurobiological aspects. On this note, future studies on paternal care and neurobiology of fatherhood among same-sex parents could adopt a multidimensional assessment of paternal involvement based on continuous scores, in order to capture the wide range of variability of the construct by using dimensional rather than dichotomous categorical outcomes (e.g., primary vs. secondary caregivers). Overall, more efforts should be put into the examination of the caregiving network in male same-sex parents, with the aim to confirm and extend preliminary findings on the influence of childcare experiences on brain activations. Remarkably, much research on male same-sex parents might constitute a fascinating perspective shedding light on the adaptability of fathers brain when the primary caregiving role is assumed and no changes associated with gestation are experienced.

## Structural Barriers for Male Same-Sex Couples Becoming Parents

Beyond the relevant benefits of conducting studies on diverse family forms, the difficulties in recruiting male same-sex parents should be considered. Even though socio-cultural changes are leading to more variation in the family structures, the access to reproduction technology and adoption as well as the legal barriers are still conspicuous challenges for the long journey to parenthood. Moreover, as a part of a marginalized community, same-sex families of men may be victim of stigmatization and contextual stressors, as they may negotiate a multiminority status for potentially being gay and being homosexual parents (Armesto, 2002). In addition, as compared to female same-sex parents, they may suffer from the importance placed on motherhood and the general devaluation of fatherhood. In this regard, studies should take into account the role of stigma-related stressors in order to isolate their effects from other factors (Farr and Vázquez, 2020).

In spite of the difficulties, we suggest that collecting more evidence about new family forms could provide valuable insights for parenting research by adding a remarkable piece of knowledge to the field. Thereby, practice and policy could be driven by emerging evidence to reduce stigma toward same-sex parents. Methodologically speaking, researchers could rule out the effect of potential existing sex roles in different-sex couples by studying a broad range of family constellations, in which the division of care for infants is far less gendered. On this note, the real effect of involvement in childcare in modulating the neurobiological responses to infant cues could be detected, thus shedding light on the effective role played by direct experience in fathering on the paternal neural activations. In conclusion, the study of neural parental caregiving network in male same-sex parents is a potential unique opportunity to examine the contribution of contextual factors such as parental role definitions, involvement in childcare, and cultural beliefs in shaping neurobiological bases and behavioral responses underlying nurturing behaviors.

## Author Contributions

MGi and MGe drafted the manuscript. SD, PR, AS, and PV substantially contributed to the conception of the manuscript and revised it critically for important intellectual content. All the authors made a direct and intellectual contribution to the work, and approved it for publication.

## Funding

This work was funded by PRIN 2017—Research Project of National Relevance—Ministry of Education, University and Research—same-sex and different-sex parent families through assisted reproduction: parenting, attachment, child adjustment and neural correlates.

## Conflict of Interest

The authors declare that the research was conducted in the absence of any commercial or financial relationships that could be construed as a potential conflict of interest.

## Publisher's Note

All claims expressed in this article are solely those of the authors and do not necessarily represent those of their affiliated organizations, or those of the publisher, the editors and the reviewers. Any product that may be evaluated in this article, or claim that may be made by its manufacturer, is not guaranteed or endorsed by the publisher.
